# Study of the Repellent Activity of 60 Essential Oils and Their Main Constituents against *Aedes albopictus*, and Nano-Formulation Development

**DOI:** 10.3390/insects13121077

**Published:** 2022-11-22

**Authors:** Weifeng Wu, Yu Yang, Yingmiao Feng, Xiaofei Ren, Yuling Li, Wenjiao Li, Jietong Huang, Lingjia Kong, Xiaole Chen, Zhongze Lin, Xiaohui Hou, Longlai Zhang, Yajie Chen, Zhaojun Sheng, Weiqian Hong

**Affiliations:** 1 School of Biotechnology and Health Sciences, Wuyi University, Jiangmen 529020, China; 2 The Third Clinical Medical College, Guangzhou University of Chinese Medicine, Guangzhou 510405, China; 3 Faculty of Southern Medicine, Guangdong Jiangmen Chinese Medicine College, Jiangmen 529000, China; 4 Department of Chemistry, Hong Kong Baptist University, Kowloon Tong, Hong Kong SAR, China; 5 International Healthcare Innovation Institute (Jiangmen), Jiangmen 529020, China; 6 School of Preclinical Medicine, Zunyi Medical University, Zunyi 563003, China; 7 MHOME (Guangzhou) Industrial Co., Ltd., Guangzhou 510700, China; 8 Department of Chemistry, University of Liverpool, Liverpool L69 7ZD, UK

**Keywords:** essential oil, repellent, *Aedes albopictus*, nanoemulsion

## Abstract

**Simple Summary:**

Due to the environment and human health concerns of synthetic repellents, essential oils (EOs) as natural alternatives have received increased attention. In this study, the repellent activity of 60 commercial EOs against *Aedes albopictus* was evaluated. In the initial screening, 8 active EOs including cinnamon, marjoram, lemongrass, bay, chamomile, jasmine, peppermint2, and thyme were selected. Twenty-one major constituents (>5% relative area) in the 8 active EOs were identified via gas chromatography-mass spectrometry (GC-MS) analysis. Cinnamaldehyde, citral and terpinene-4-ol displayed the highest repellent activity with more than 60% RR, which were more active than *N*,*N*-diethyl-3-methyl benzoyl amide (DEET). Next, their nanoemulsions were prepared and characterized. In the arm-in-cage assay, cinnamaldehyde- and citral-based nanoemulsions have prolonged mosquito protection time compared with their normal solutions.

**Abstract:**

Mosquitoes are one of the most important disease vectors from a medical viewpoint in that they transmit several diseases such as malaria, filariasis, yellow and Dengue fever. Mosquito vector control and personal protection from mosquito bites are currently the most efficient ways to prevent these diseases. Several synthetic repellents such as DEET, ethyl butylacetylaminopropionate (IR3535) and 1-(1-methylpropoxycarbonyl)-2-(2-hydroxyethyl)piperidine) (Picaridin), have been widely used to prevent humans from receiving mosquito bites. However, the use of synthetic repellents has raised several environment and health concerns. Therefore, essential oils (EOs) as natural alternatives receive our attention. In order to discover highly effective mosquito repellents from natural sources, the repellent activity of 60 commercial EOs against *Ae. albopictus* was screened in this study. Eight EOs including cinnamon, marjoram, lemongrass, bay, chamomile, jasmine, peppermint2, and thyme, showed a suitable repellent rate (>40%) at the tested dose of 10 μg/cm^2^. Then, their main constituents were analyzed by GC-MS, and the active constituents were identified. The most active compounds including cinnamaldehyde, citral and terpinen-4-ol, exhibited an 82%, 65% and 60% repellent rate, respectively. Moreover, the nanoemulsions of the three active compounds were prepared and characterized. In the arm-in-cage assay, the protection times of the nanoemulsions of cinnamaldehyde and citral were significantly extended compared with their normal solutions. This study provides several lead compounds to develop new mosquito repellents, and it suggests that nanoemulsification is an effective method for improving the duration of the activity of natural repellents.

## 1. Introduction

Mosquito bites not only cause several allergic reactions including itching and swelling, but are also a mechanism to transmit pathogens between people and animals [1]. *Ae. albopictus* (Skuse), also known as Asian tiger mosquito, is a key carrier of dengue, Zika and yellow fever viruses [2]. Mosquito control and personal protection from mosquito bites are currently the most important measures to prevent these diseases [3]. One of the most common approaches is to use insect repellents. Insect repellents can be classified into two categories: spatial and contact repellents. Their action modes are different. Spatial repellents, such as some synthetic pyrethroids and botanical compounds, are generally highly volatile and capable of diffusing through the air in treated regions [4]. The repellent vapors from spatial repellents lead to the aversive behavior and deleterious physiological response of host-seeking mosquitoes [5,6]. Contact repellents such as DEET, picaridin, and IR3535, are capable of attenuating the antennal responses of mosquitoes to various human and veterinary attractive odorants via direct inhibition or attenuation of action potential amplitudes or frequencies emanating from olfactory receptor neurons [6]. Nowadays, the most used synthetic repellents represented by DEET are contact repellents, which need to be applied directly on the human skin. The use of synthetic repellents to control mosquitoes raises several concerns related to the environment and human health [7]. On the contrary, natural essential oils (EOs) have advantages such as the wide spectrum of efficacy against mosquitoes, multiple mode of actions, low residue and low toxicity [8,9], thereby they have received our attention.

EOs are defined as volatile oils that have strong aromatic components and give a distinctive odor, flavor or scent to an aromatic plant [10]. Many natural EOs with the function of repelling mosquitoes have been discovered and exploited [7,11]. Among them, citronella oil was the most widely used before the 1940s, and is still used today in many formulations [12]. Others include eucalyptus, clove, lavender and lemon oils [3,13]. In modern times, the development of natural repellents was neglected after the appearance and rapid development of synthetic repellents. However, with increasing attention and demand for healthy and environmentally friendly mosquito repellents, the research and development of natural EOs as mosquito repellents have begun to speed up again [14]. In our previous study, the larvicidal activity and synergistic effect with deltamethrin of 53 commercial EOs were screened and studied [15]. In order to discover highly effective mosquito repellents from natural sources and to investigate the further applications of EOs in mosquito-vector control, the repellent activity of 60 commercial EOs against *Ae. albopictus* was evaluated in this study. The active constituents of the active EOs were identified. Moreover, the nanoformulation was used to improve the duration of natural repellents.

## 2. Materials and Methods

### 2.1. Essential Oils and Reagents

The information of 60 EOs is listed in Table S1 of supporting information. Lemon eucalyptus EO was purchased from Jiangxi Hualong Plant Flavor Co., Ltd., Ji’an, China; and other EOs were purchased from Guangzhou Yuxitang Cosmetics Co., Ltd. Guangzhou, China. All were pure EOs with no additive.

Benzyl acetate (purity: 95%), 2,2,4,6,6-pentamethyl-heptane (98%), menthol (97%), terpinen-4-ol (95%), cinnamaldehyde (98%), citral (isomers of neral and geranial, 98%), *p*-menthone (*cis*-&*trans*-isomers, 98%) and diethyl phthalate (99%) were purchased from Bidepharm (Shanghai, China). *p*-Cymene (97%) was purchased from Energy Chemical (Shanghai, China). Limonene (95%), linalool (96%), thymol (99%), carvacrol (98%), eugenol (99%), and *β*-caryophyllene (90%) were purchased from TCI (Shanghai, China). *γ*-Terpinene (95%), PEG-40 (HLB value: 13-14; pH value: 5.0-7.0), 1,2-propanediol (99%) were purchased from Macklin (Shanghai, China). *α*-Terpineol (96%) was purchased from Alfa Aesar (UK). (*E*)-2-Hexyl- cinnamaldehyde (92%) and DEET (99%) were purchase from Aladdin (Shanghai, China). Diisobutyl phthalate (99%) was purchased from J&K Scientific (Beijing, China). Defibrinated sheep blood was purchased from Rigorous Scientific (Guangzhou, China).

### 2.2. Mosquitoes

The *Ae. albopictus* larvae were collected from Jiuwei Village, Huangpu District of Guangzhou of China (latitude: 23°06′24.17″ N, longitude: 113°26′43.07″ E) in March 2013. The adult mosquitoes that emerged from these larvae were identified as *Ae. Albopictus* according to the morphology. The mosquitoes were reared in the insectary of the International Healthcare Innovation Institute (Jiangmen) of China. The insectary was kept at 26 ± 2 °C, 70 ± 5% relative humidity with a photoperiod of 14 h light and 10 h dark. Larvae were fed daily with fish food, and adults were fed with 5% glucose solution. 5 to 7-day-old female mosquitoes were used in the bioassays.

### 2.3. Repellent Activity Bioassay

**Figure 1:**
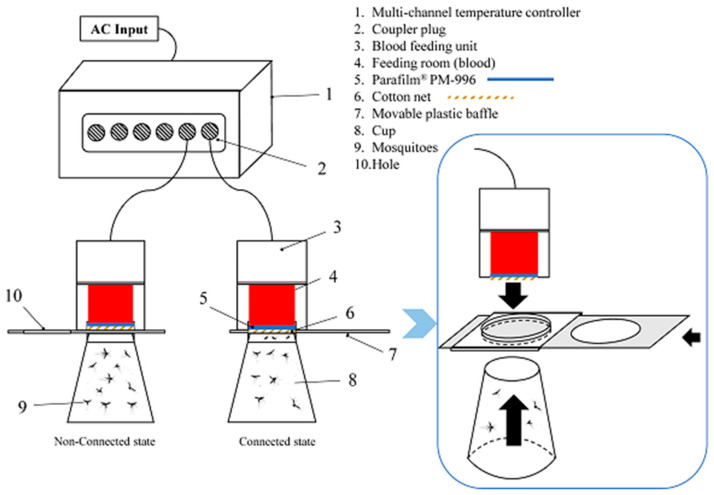
Repellent activity test device.

The repellent activity of 60 EOs was tested using the Hemotek membrane feeding system (Discovery Workshops, Accrington, UK), and following the reported method [16,17], with some modification. As shown in Figure 1, 2.5 mL of sterile defibrinated sheep blood was placed in the feeding room. The blood was preheated and kept at 37.5 °C to simulate the blood temperature of human beings. Parafilm was used to seal the blood and simulate the skin. The exposed surface was 15.9 cm^2^. 159 μg repellent was dissolved in acetone, giving the testing solution. The solution was uniformly loaded on 15.9 cm^2^ of cotton net, and then the net was dried at room temperature for 10 min to evaporate the acetone. Next, the net was fixed and covered on the parafilm. The same cotton net loaded with pure acetone was used in the control group.

When the bioassay began, 20 to 30 female mosquitoes were put in a disposable cup without a bottom. The cup was sealed with a movable plastic baffle, which controlled the exposure status of mosquitoes to repellent and blood. Female mosquitoes were exposed for 30 min. After that, the test mosquitoes were immobilized using CO_2_, and transferred to the fridge to freeze. Then, the numbers of blood-fed and unfed mosquitoes were recorded. The repelling rate (RR) was calculated using Equation (1) [17]. Three replicates were carried out for each sample, and the average RR was calculated.

(1) $$\mathrm{RR}\;(\%)=\frac{F_{0}/T_{0}-F/T}{F_{0}/T_{0}}\;\times \;100\%$$

where *F_0_* and *T_0_* are the numbers of fed mosquitoes and all mosquitoes in the control group, respectively. *F* and *T* are those in the test group.

### 2.4. Chemical Compositions Analysis

The chemical compositions of EOs were analyzed using GC-MS (Thermo Scientific, TRACE 1300 GC coupled to an ISQ Qd Mass Spectrometer and equipped with a TG-5 MS capillary column). The analysis method used follows that of Sheng et al. [15]. Based on the search results of National Institute of Standards and Technology (NIST) mass spectral data library, the constituents were identified through Kovats retention indices (RI) combined with the comparisons of commercially available standards.

### 2.5. Nanoemulsion Preparation and Characterization

The nanoemulsion was prepared by slightly modifying the reported procedures [18,19]. It was composed of 20% (*w*/*v*) active constituent, 40% (*w*/*v*) cosurfactants (1,2-propanediol/ethanol = 1/1), 20% (*w*/*v*) PEG 40 as an emulsifier, and 20% (*w*/*v*) water. First, 20% (*w*/*v*) of the active constituent was added to the emulsifier, and then stirred at 200 rpm to give the oil phase. Next, the aqueous phase consisting of 1,2-propanediol, ethanol and water were added dropwise to the oil phase mixtures. Oil and aqueous phases were mixed evenly through stirring with a rate of 500 rpm at room temperature (26 °C) for 1 h.

The particle size and polydispersity index (PDI) values of the nanoemulsions were measured with a modular dynamic light scattering (DLS) system (Microtrac Inc, Dusseldorf, Germany). Moreover, the structure and morphology of cinnamaldehyde-based nanoemulsion was characterized by transmission electron microscopy (TEM, JEM-1400 PLUS, JEOL Ltd., Tokyo, Japan).

### 2.6. Repellent Longevity Bioassay

The repellent longevity of three EOs nanoemulsions and their normal solutions were evaluated using arm-in-cage assay. Referred to the methods of Logan et al. [20], 50 female mosquitoes which had been starved for 12 h and had not a blood meal, were used in each cage (30 × 30 × 30 cm). Six human volunteers (male/female = 3/3) with no or little allergic reaction to bites were selected for the trials.

Before the trials, the hands of volunteers were cleaned with water, followed by 75% ethanol. First, 62.5 μL of 20% (*w*/*v*) EO nanoemulsion or normal solution in ethanol, was applied to a volunteer’s hand with the exposure area of 25 cm^2^. The other part of the hand was covered. When evaluated with the nanoemulsion, the nanoemulsion without EO served as a negative control on the other hand. When evaluated with the normal solution, ethanol alone served as a negative control on the other hand. During the test, the control hand was firstly inserted into the cage to confirm the aggressivity of mosquitoes. Then the treated hand was inserted into the same cage and kept for 2 min. If any mosquitoes landed on the hand, it meant the loss of the repellent protection. The protection time of each volunteer was recorded, and the average time of six volunteers was calculated.

The work was carried out in accordance with the Code of Ethics of the World Medical Association (Declaration of Helsinki) for experiments involving humans.

### 2.7. Statistical Analysis

The droplet sizes of nanoemulsions were expressed as mean ± standard deviation of three replicates. In the stability study, the drop size changes of nanoemulsions at 4 °C and 25 °C within 28 days were calculated and processed by Graphpad Prism 8.0 (San Diego, CA, USA). The multi-T test was used to analyze the stability between different groups, and compare the repellent longevity between EO nanoemulsion and its normal solution. The results were considered statistically significant when *p* < 0.05.

## 3. Results

### 3.1. Screen of Repellent Activity of 60 EOs Using Membrane Feeding Device

The repellent activities of 60 EOs at the dosage of 10 μg/cm^2^ exposed for 30 min against female adults were screened. Results are shown in Table 1 and Table S2. The most effective EO was cinnamon with 77% RR, which was even higher than DEET in our assays. Seven other EOs such as marjoram, lemongrass, bay, chamomile, jasmine, peppermint2, and thyme resulted in more than 40% RR, indicating high repellent activity. Eighteen EOs including osmanthus, myrrh and melissa, displayed moderate repellent activity with 20–36% RR. Fourteen EOs including clove, michelia alba flower, and basil, showed poor repellent activity with 10–19% RR. Twenty other EOs showed very low or no repellent activity, and some EOs even had attractive action to *Ae. Albopictus*. DEET as a positive control showed 59 ± 3% RR under the same conditions.

### 3.2. Chemical Compositions of 8 Active EOs

There were 8 EOs with more than 40% RR. Among them, the main constituents of lemongrass and thyme has been reported by our team [15]. The chemical compositions of 6 other EOs were analyzed by GC-MS in this study. In addition to comparing the retention index (RI) values with the reported data, the main components with relative area (RA) > 5% were finally identified via comparison with commercially available standards. In total, 21 main components (RA > 5%) were identified in the 8 active EOs.

As shown in Table 2, cinnamon EO was dominated by cinnamaldehyde (82.6%). The main components of marjoram EO included three monoterpenes and two terpenols. The chief component was terpinen-4-ol (32.6%), followed by *α*-terpineol (12.4%), *γ*-terpinene (10.9%), limonene (9.4%) and *p*-cymene (7.1%). The constituents of bay EO were relatively diverse. Eugenol (36.3%) and *β*-caryophyllene (36.7%) were the two main constituents. Besides, cinnamaldehyde (6.4%) and diisobutyl phthalate (5.6%) also had important contributions. There were only two constituents accounted for 5% in chamomile EO. The highest constituent was diethyl phthalate (43.0%), followed by limonene (12.6%). The major constituents of jasmine EO were linalool (30.2%), followed by (*E*)-2-hexyl-cinnamaldehyde (25.3%) and benzyl acetate (17.1%). Menthol (30.9%) and the isomer mixtures of *p*-menthone (26.8%) were the most noticeable constituents of peppermint2 EO, followed by 2,2,4,6,6-pentamethyl-heptane (22.4%) and limonene (6.8%).

### 3.3. Repellent Activity of 21 Main Constituents from 8 Active EOs

**Figure 2:**
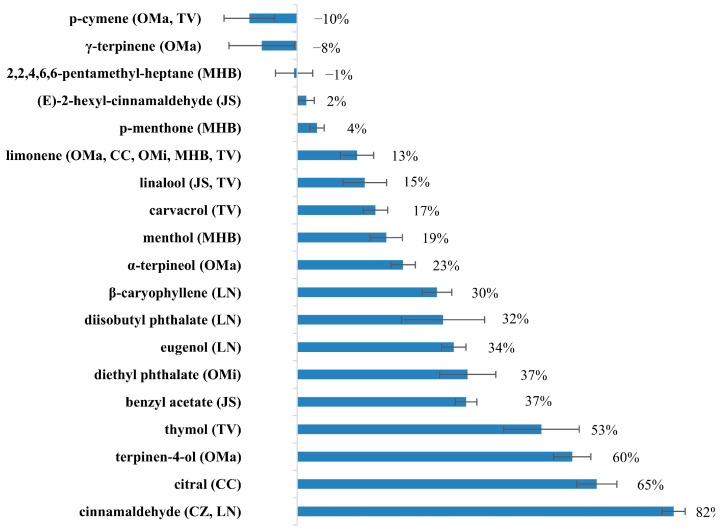
Repellency rate of the main constituents of the 8 most active EOs.

**Figure 3:**
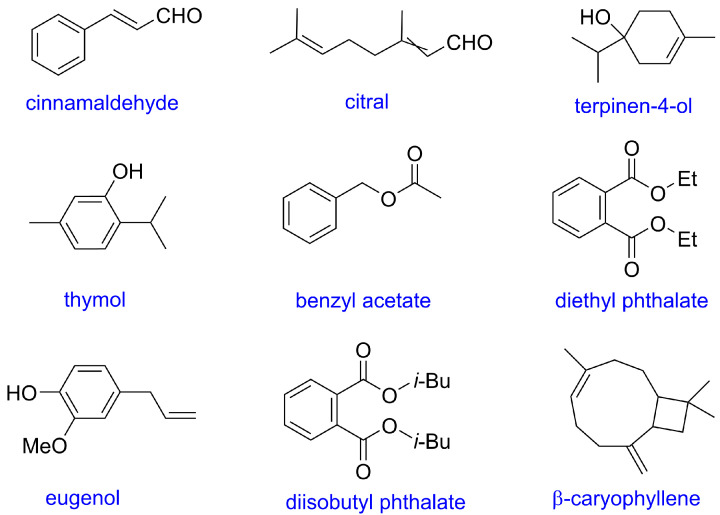
Chemical structures of 9 active constituents from 8 active EOs.

The repellent activity of all main constituents of the 8 active EOs was tested. The RR of these compounds were shown in Figure 2 and Table S3. Cinnamaldehyde showed the highest repellent activity with 82% RR, followed by citral (isomers mixture of neral and geranial) with 65% RR, terpinen-4-ol with 60% RR, and thymol with 53% RR. Benzyl acetate, diethyl phthalate, eugenol, diisobutyl phthalate, and *β*-caryophyllene performed moderated repellent activity with >30% RR. The chemical structures of these 9 active compounds were depicted in Figure 3.

Besides, *α*-terpineol, menthol, carvacrol, linalool, and limonene had some repellent activity with 13-23% RR. (*E*)-2-hexyl-cinnamaldehyde and *p*-menthone (mixture of *cis*- and *tran*s-isomers) showed very low repellent activity. Three compounds including 2,2,4,6,6-pentamethyl-heptane, *γ*-terpinene, and *p*-cymene even attract mosquitoes compared with the negative control. Therefore, three compounds (cinnamaldehyde, citral and terpinen-4-ol) with higher RR than DEET, were selected as the objectives in the following formulation study.

### 3.4. Characterization of Nanoemulsions of Three Active Compounds

**Figure 4:**
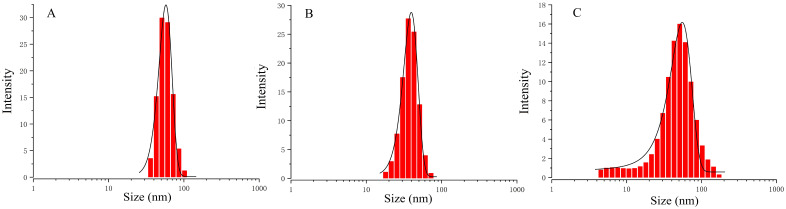
Droplet size distribution of three nanoemulsions ((**A**) cinnamaldehyde; (**B**) citral; (**C**) terpinen-4-ol).

**Figure 5:**
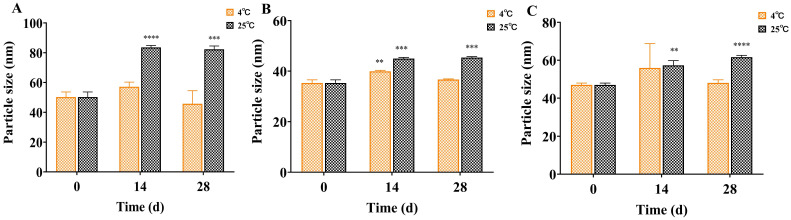
Particle size changes of three nanoemulsions ((**A**) cinnamaldehyde; (**B**) citral; (**C**) terpinen-4-ol) for 28 days at 4 °C and 25 °C (compared with 0 day, ** means *p* ≤ 0.01; *** means *p* ≤ 0.001; **** means *p* ≤ 0.0001).

The droplet sizes of the nanoemulsions of cinnamaldehyde, citral and terpinen-4-ol were determined using DLS. As shown in Figure 4, the average particle sizes of freshly prepared samples were 50.2 ± 3.4 nm (cinnamaldehyde), 35.3 ± 1.8 nm (citral), and 47.0 ± 1.0 nm (terpinen-4-ol), with PDI values of 0.041, 0.057, and 0.180, respectively. In addition, the stability of the three nanoemulsions was monitored under 4 °C and 25 °C. As shown in Figure 5, the particle sizes of the three nanoemulsions remained stable with storage at 4 °C. No phase separation or other visual changes occurred during 28 days. However, a relative destabilization of the nanoemulsions appeared at 25 °C after 28 days storage. Therefore, these nanoemulsions should be stored at 4 °C.

**Figure 6:**
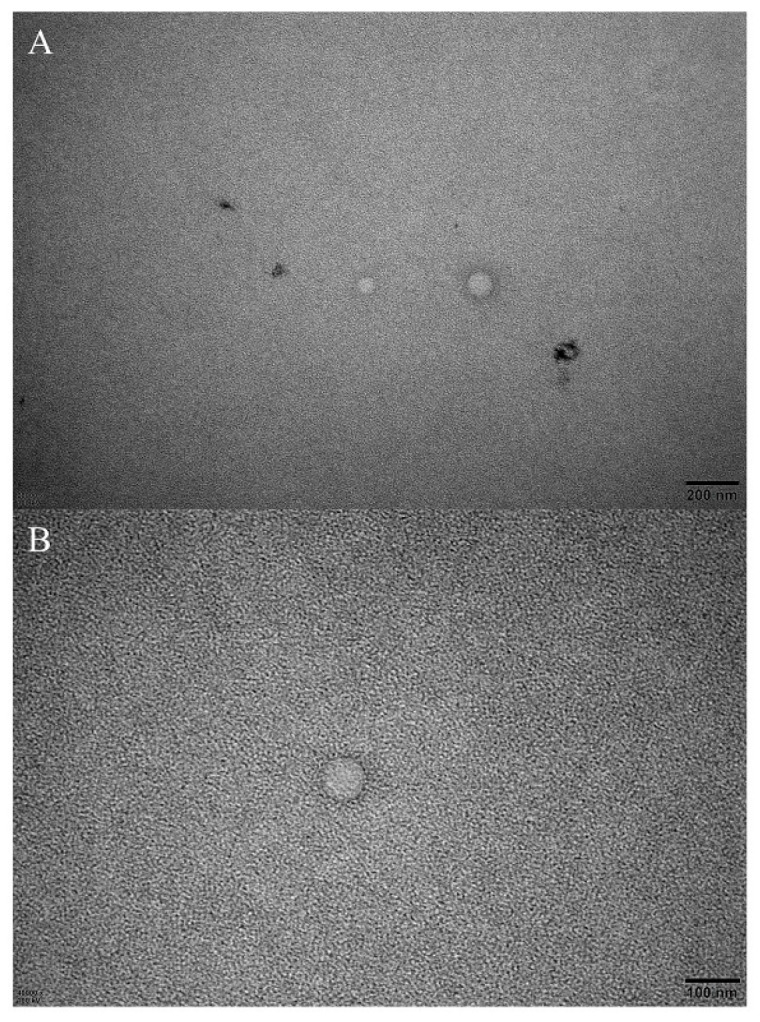
Droplet of cinnamaldehyde nanoemulsion under TEM. ((**A**) × 20,000 magnification; (**B**) × 40,000 magnification).

Moreover, the structure and morphology of the nanoemulsion of cinnamaldehyde were analyzed by TEM. As shown in Figure 6, phosphotungstic acid-stained cinnamaldehyde droplets were clearly visible and spherical. The droplet size in TEM was similar to that measured by DLS.

### 3.5. Repellent Longevity Comparison

The protection times of three nanoemulsions at the dosage of 500 μg active constituent per cm^2^ against *Ae. albopictus* were evaluated, and compared with the same dosage of their normal solutions. As shown in Table 3, except for terpinen-4-ol, the repellent longevity of other two nanoemulsions was significantly extended (*p* < 0.05). For example, the protection time of the nanoemulsion of cinnamaldehyde was 146 min, which was nearly 1 h longer than its normal solution.

## 4. Discussion

### 4.1. Screening of Repellent Activity of 60 EOs

Nowadays, EOs have been widely used for bactericidal [21], fungicidal [22], acaricidal [23,24], insecticidal [25,26,27,28], medicinal and cosmetic applications [10]. Moreover, EOs as natural insect repellents have a long usage history in China and Arab countries, thereby being an ideal resource to discover new natural repellents [7,14,29,30]. Normally, the repellent activity of EOs against mosquitoes is evaluated using an arm-in-cage assay [31,32], which needs a certain number of qualified volunteers. Thus, extensive screening is difficult and of low efficiency. Thanks to our modified Hemotek membrane feeding system, the repellent activity of up to 60 EOs against *Ae. albopictus* was screened in this study. As shown in Table 1, the RRs of cinnamon, marjoram and lemongrass EOs at 10 μg/cm^2^ exposed for 30 min were 77 ± 4%, 57 ± 2%, and 54 ± 3%, which were comparable to that of DEET. The application of cinnamon and lemongrass EOs in the mosquito-vector control have been widely reported [33]. Pohlit et al. [34] reviewed the patent literatures on mosquito repellent inventions which contain plant essential oils. Cinnamon and lemongrass EOs were each cited in >10% of patents. Peach et al. [35] reported cinnamon bark and lemongrass EOs are effective at spatially repelling *Ae. aegypti* in field settings. Our resutlts suggested that the two EOs also had strong contact repellent activity against mosquitoes. The reports on the repellent activity of marjoram EO against mosquitoes were relatively few, except Kang et al. [36] reported that majoram EO showed good repellency (repellent efficacy > 60%) at a concentration of 5 μg/cm^2^ against Culex pipiens pallens. Our results also supported that majoram EO had potent repellent activity against *Ae. albopictus*. It is worth noting that citronella ceylon and lemon eucalyptus EOs are well-known spatial mosquito repellents [34,37]. They repel mosquitoes via diffusing volatile odors that can affect mosquitoes’ behavior in human host detection. However, in our assay, their contact RRs were only 29% and 11%, respectively. The results suggest that there were some differences between the contact and spatial repellent activities of the same EO, and this difference maybe come from different action modes [38].

### 4.2. Identification of Active Constituents

The chemical compositions and their contents in EOs were largely impacted by the origin, the extraction method, extraction part and other factors [39]. The efficacy of EOs depends on the chemical compositions. Therefore, the active ingredients need to be identified. The GC-MS analysis results of 8 active EOs with more than 40% RR are shown in Table 2. There were 21 compounds accounted for more than 5% RA in these 8 active EOs. The main constituents include terpenes, oxidized terpenes (including alcohols, aldehyde and ketone), aldehydes, esters and others.

The repellent activities of 21 main constituents are shown in Figure 2. The high repellent activity of cinnamon EO mainly came from that of cinnamaldehyde. The main active constituent of marjoram EO was terpinen-4-ol. Citral as the mixture of neral and geranial isomers was the main active ingredient of lemongrass EO. The high repellent activity of bay EO was caused by the combined action of cinnamaldehyde, eugenol, diisobutyl phthalate, and β-caryophyllene. The major active constituents of chamomile and jasmine EOs were diethyl phthalate and benzyl acetate, respectively. Thymol had a major contribution to the potent activity of thyme EO. In addition, carvacrol, linalool, and limonene also had some contributions. Peppermint2 EO performed potent repellent activity in the initial screening. However, its main constituents including p-menthone, menthol, and limonene, only showed moderate or low repellent activity. It indicated that some minor constituents (<5% RA) might have an important effect on the repellent activity; that is to say, a synergistic phenomenon among these constituents may result in a higher bioactivity compared to the isolated components [7].

The chemical structures of 9 active compounds with more than 30% RR are depicted in Figure 3. Their structures were diverse, thereby it was difficult to find some common features among them. However, their RI values were relatively big in the GC-MS analysis. Conversely, the compounds with lower RI values such as 2,2,4,6,6-pentamethyl-heptane, p-cymene, and γ-terpinene, displayed low repellent activity. It indicated that the physicochemical properties like volatility greatly affected the repellent activity [7].

### 4.3. Nano-Formulation Study

Some plant-derived repellents are comparable to, or even better than synthetics; however, EO repellents tend to being short-lived in their effectiveness due to their high volatility [7,40]. At present, the use of nanotechnology to develop new formulations is one of the most important ways to slow the release rate and thus prolong the protection time [37,41,42,43]. In our repellent assay, three compounds including cinnamaldehyde, citral, and terpinen-4-ol, showed higher repellent activity than DEET when they were exposed for 30 min in the mosquito cage, which suggested that they have potential for further development as new mosquito repellents.

In our study, based on water as the aqueous phase, the active constituents (cinnamaldehyde or citral or terpinen-4-ol) as the oil phase, PEG 40 as the surfactant, and 1,2-propanediol/ethanol = 1/1 (*w*/*v*) as the cosurfactants, the nanoemulsions were prepared by a low-energy method. The nanoemulsions were characterized by particle size, PDI value and morphological image (cinnamaldehyde), which suggests the physicochemical characterizations of these nanoemulsions were desirable. In order to investigate the stability of these nanoemulsions, the particle sizes were monitored for 28 days (Figure 5). The results showed that the temperature and storage time affected the nanoemulsion stability. The droplet sizes increased in 28 days at 25 °C storage. However, no statistically significant difference was observed in droplet sizes for 28 days at 4 °C, where the *p*-value was >0.05. It suggested that these nanoemulsions have good stability at 4 °C, which was in agreement with our previous study [44].

Next, the protection times of these nanoemulsions were evaluated and compared with the normal solutions. As shown in Table 3, the protection times of the nanoemulsions of cinnamaldehyde and citral were greatly extended. It suggests that the active constituents were released more slowly in the nanoformulations [19]. Drapeau et al. [45] also reported that the microemulsion of p-menthane-diol obtained an extension of the protection time against *Ae. aegypti*. Nuchuchua et al. [46] reported that the nanoemulsions of citronella oil, hairy basil oil and vetiver oil, with droplet sizes ranging from 150 to 220 nm, had prolonged mosquito protection time. It is worth noting that the RRs of cinnamaldehyde (82%) and citral (65%) were higher than the positive reference DEET (59%); however, their protection times were much shorter than that of DEET, which was probably due to the high volatility and the physicochemical instability of the two natural compounds. Our results suggest that nano-formulations could be used to alleviate this problem. Rehman et al. [13] reviewed the protection times of essential oils against mosquitoes. The cinnamaldehyde-based nanoemulsion displayed a longer protection time (146 min) than most of the reported natural repellents.

## 5. Conclusions

In conclusion, the mosquito repellent activity of up to 60 commercial EOs at the dosage of 10 μg/cm^2^ exposed for 30 min were screened. Eight EOs including cinnamon, marjoram, lemongrass, bay, chamomile, jasmine, peppermint2, and thyme, displayed potent repellent activity with more than 40% RR. Twenty-one major constituents (>5% RA) in the 8 active EOs were identified via GC-MS analysis. Cinnamaldehyde, citral and terpinen-4-ol displayed the highest repellent activity with more than 60% RR. Their nanoemulsions were prepared and characterized. In the arm-in-cage assay, cinnamaldehyde- and citral-based nanoemulsions have a prolonged mosquito protection time compared with their normal solutions. This study not only discovered several leading compounds from which to develop novel plant-derived mosquito repellents, but also suggested that the nano-formulations could improve the duration of natural repellents. However, the repellent mechanism study is lacking. More in-depth studies on the action mode and chemical structural modifications remain to be explored in the future. Meanwhile, the combination of different plant-derived repellents together will be investigated to improve their efficacies against mosquitoes. Moreover, the nano-formulation needs to be further optimized to improve the stability of the nanoemulsions stored at room temperature. These works are in progress in our lab, and will be reported in due course.

## Figures and Tables

**Table 1:** Repelling rate of 60 EOs at 10 μg/cm^2^ exposed for 30 min.

EO	RR (%) Mean ± SD	EO	RR (%) Mean ± SD
Cinnamon	77 ± 4%	Cedarwood	16 ± 9%
Marjoram	57 ± 2%	Palmarosa	15 ± 2%
Lemongrass	54 ± 3%	Michelia alba leaf	15 ± 5%
Bay	51 ± 1%	Lime	14 ± 3%
Chamomile	47 ± 3%	Rosemary	13 ± 2%
Jasmine	44 ± 4%	Parsley	13 ± 8%
Peppermint2	42 ± 10%	Juniper	12 ± 6%
Thyme	41 ± 2%	Lemon eucalyptus	11 ± 2%
Osmanthus	36 ± 5%	Capsicum	10 ± 5%
Myrrh	33 ± 1%	Angelica	9 ± 4%
Melissa	33 ± 5%	Cajeput	9 ± 4%
Grapefruit	31 ± 3%	Orange sweet	8 ± 3%
Sandalwood	31 ± 4%	Carrot seed	8 ± 5%
May Chang	30 ± 2%	Eucalyptus	6 ± 6%
Citronella ceylon	29 ± 3%	Tangerine	5 ± 0%
Nutmeg	29 ± 3%	Violet	3 ± 3%
Vetiver	28 ± 3%	Fennel	3 ± 3%
Ay tsao	26 ± 5%	Rose	3 ± 10%
Bergamot	25 ± 8%	Ginger	2 ± 4%
Clary sage	25 ± 8%	Ylang ylang	1 ± 2%
Petitgrain	24 ± 2%	Chinese ilex	1 ± 5%
Green tea	23 ± 4%	Cypress	0 ± 1%
Geranium	23 ± 4%	Black pepper	−1 ± 10%
Verbena	22 ± 6%	Cumin	−2 ± 3%
Benzoin	21 ± 2%	Pine fir	−5 ± 6%
Peppermint1	20 ± 3%	Mandarin	−6 ± 1%
Clove	19 ± 3%	Rosewood	−7 ± 2%
Michelia alba flower	19 ± 4%	Frankincense	−10 ± 10%
Basil	18 ± 7%	Lemon	−12 ± 7%
Patchouli	17 ± 3%	DEET (positive control)	59 ± 3%
Neroli	17 ± 5%		

**Table 2:** Main chemical constituents of 8 active EOs.

No	Component ^a^		RI ^b^	RI lit. ^c^	CZ ^d^	Oma ^d^	CC ^d^ [15]	LN ^d^	OMi ^d^	JS ^d^	MHB ^d^	TV ^d^ [15]
	Name	CAS No.										
1	2,2,4,6,6-pentamethyl-heptane	13475-82-6	991	995							22.4	
2	*p*-cymene	99-87-6	1025	1026		7.1						17.1
3	limonene	138-86-3	1029	1031		9.4	10.7		12.6		6.8	12.1
4	*γ*-terpinene	99-85-4	1059	1062		10.9						
5	linalool	78-70-6	1101	1101						30.2		6.9
6	*trans*-*p*-menthone	89-80-5	1156	1154							15.9	
7	benzyl acetate	140-11-4	1166	1165						17.1		
8	*cis*-*p*-menthone	491-07-6	1167	1165							10.9	
9	menthol	89-78-1	1175	1173							30.9	
10	terpinen-4-ol	562-74-3	1179	1182		32.6						
11	*α*-terpineol	98-55-5	1192	1189		12.4						6.9
12	neral	106-26-3	1242	1242			34.2					
13	geranial	141-27-5	1271	1273			35.4					
14	cinnamaldehyde	104-55-2	1272	1278	82.6			6.4				
15	thymol	89-83-8	1294	1292								30.6
16	carvacrol	499-75-2	1303	1300								8.7
17	eugenol	97-53-0	1359	1356				36.3				
18	*β*-caryophyllene	87-44-5	1426	1428				36.7				
19	diethyl phthalate	84-66-2	1598	1594					43.0			
20	(*E*)-2-hexyl-cinnamaldehyde	101-86-0	1759	1749						25.3		
21	diisobutyl phthalate	84-69-5	1875	1877				5.6				
Other components					17.4	27.6	19.7	15.0	44.4	27.4	13.1	17.7

^a^ Components are listed in the order of RI value. Only major components (RA > 5%) are listed in the table. ^b^ Linear retention index on TG-5MS column, experimentally determined using homologous series of C_8_-C_30_ alkanes. ^c^ Linear retention index taken from https://webbook.nist.gov/chemistry/ (accessed on 18 July 2022). ^d^ CZ: *Cinnamomum zeylanicum* (cinnamon); OMa: *Origanum marjorana* (marjoram); CC: *Cymbopogon citratus* (lemongrass); LN: *Laurus nobilis* (bay); OMi: *Ormenis mixta* (Chamomile); JS: *Jasminum sambac* (Jasmine); MHB: *Mentha haplocalyx Briq* (peppermint); TV: *Thymus vulgaris* (thyme).

**Table 3:** Comparison between the protection times of EO nanoemulsion and its normal solution.

Compounds	Average Protection Time (min)		*p* Value ^a^
	Normal Solution	Nanoemulsion	
cinnamaldehyde	94 ± 14	146 ± 19	0.004, **
citral	49 ± 14	83 ± 15	0.017, *
terpinen-4-ol	23 ± 9	26 ± 14	0.670, ns
DEET (positive control)	>360	>360	

^a^ ns (not significant) means *p* > 0.05, * means *p* < 0.05, ** means *p* < 0.01.

## Supplementary Materials

The following supporting information can be downloaded at: https://www.mdpi.com/article/10.3390/insects13121077/s1, Table S1: information of 60 EOs; Table S2: repelling rate of 60 EOs at 10 μg/cm^2^ exposed for 30 min; Table S3: repelling rate of the main constituents from 8 active EOs.
