# Supplementary material for: Study of the Repellent Activity of 60 Essential Oils and Their Main Constituents against Aedes albopictus, and Nano-Formulation Development
Source: Insects. 2022 Nov 22;13(12):1077. doi: 10.3390/insects13121077 (PMC9782114; doi:10.3390/insects13121077)
Supplement: Supplementary file 1 [file insects-13-01077-s001.zip › insects-1994694-supplementary.pdf]

**Table S1.** Information of 60 EOs

| No. | Name of EOs | Scientific name | Extracted part | Family | Origin |
|---|---|---|---|---|---|
| 1 | Cinnamon | *Cinnamomum zeylanicum* | bark | *Lauraceae* | Sri Lanka |
| 2 | May Chang | *Litsea cubeba* | fruit | *Lauraceae* | Malaysia |
| 3 | Black pepper | *Piper nigrum* | fruit | *Piperaceae* | Malaysia |
| 4 | Grapefruit | *Citrus paradisi* | peel | *Rutaceae* | Brazil |
| 5 | Bergamot | *Citrus bergamia* | peel | *Rutaceae* | Italy |
| 6 | Petitgrain | *Citrus aurantium* | leaf | *Rutaceae* | Italy |
| 7 | Neroli | *Citrus aurantium bigarade* | flower | *Rutaceae* | Italy |
| 8 | Lime | *Citrus medica* | peel | *Rutaceae* | Italy |
| 9 | Orange sweet | *Citrus vulgaris* | peel | *Rutaceae* | Brazil |
| 10 | Tangerine | *Citrus tangerina* | peel | *Rutaceae* | Malaysia |
| 11 | Mandarin | *Citrus reticulata* | peel | *Rutaceae* | Brazil |
| 12 | Lemon | *Citrus limonum* | peel | *Rutaceae* | Argentina |
| 13 | Cajeput | *Melaleuca quinquenervia* | leaf | *Myrtaceae* | England |
| 14 | Eucalyptus | *Eucalyptus radiata* | branch and leaf | *Myrtaceae* | Australia |
| 15 | Lemon eucalyptus | *Eucalyptus citriodora* | leaf | *Myrtaceae* | China |
| 16 | Rosewood | *Aniba Rosaeodora* | heart wood | *Lauraceae* | Brazil |
| 17 | Sandalwood | *Santalum album* | heart wood | *Santalaceae* | India |
| 18 | Cedarwood | *Cedrus atlantica* | bark and trunk | *Pinaceae* | Morocco |
| 19 | Pine fir | *Pinus sylvestris* | needle and cone | *Pinaceae* | Australia |
| 20 | Violet | *Viola odorata* | flower | *Violaceae* | France |
| 21 | Green tea | *Camellia sinensis* | leaf | *Theaceae* | Portugal |
| 22 | Carrot seed | *Daucus carota* | seed | *Apiaceae* | Hungary |
| 23 | Parsley | *Petroselinum sativum* | leaf | *Apiaceae* | China |
| 24 | Angelica | *Angelica archangelica* | seed and root | *Apiaceae* | China |
| 25 | Fennel | *Foeniculum vulgare* | seed | *Apiaceae* | Vietnam |
| 26 | Cumin | *Cuminum cyminum* | seed | *Apiaceae* | India |
| 27 | Verbena | *Lippia citriodora* | stem leaf | *Verbenaceae* | Germany |
| 28 | Nutmeg | *Myristica fragrans* | fruit | *Myristicaceae* | Indonesia |
| 29 | Geranium | *Pelargonium graveolens* | flower and leaf | *Geraniaceae* | France |
| 30 | Benzoin | *Styrax benzoin* | stem | *Styracaceae* | Vietnam |
| 31 | Capsicum | *Capsicum frutescens* | fruit | *Solanaceae* | China |
| 32 | Ginger | *Zingiber officinale* | rhizome | *Zingiberaceae* | China |
| 33 | Ylang ylang | *Cananga odorata* | flower | *Annonaceae* | Indonesia |
| 34 | Chinese ilex | *Ilex purpurea hassk* | seed | *Aquifoliaceae* | China |

| | | | | | |
|---|---|---|---|---|---|
| 35 | Palmarosa | *Cymbopogon martini* | flower and leaf | *Poaceae* | Spain |
| 36 | Rose | *Rosa rugosa* | flower | *Rosaceae* | England |
| 37 | Bay | *Laurus nobilis* | leaf | *Lauraceae* | Morocco |
| 38 | Osmanthus | *Osmanthus fragrans* | flower | *Oleaceae* | East India |
| 39 | Jasmine | *Jasminum sambac* | flower | *Oleaceae* | Morocco |
| 40 | Clove | *Eugenia caryophyllata* | flower | *Myrtaceae* | Sri Lanka |
| 41 | Michelia alba leaf | *Michelia alba DC.* | leaf | *Magnoliaceae* | China |
| 42 | Michelia alba flower | *Michelia alba DC.* | flower | *Magnoliaceae* | China |
| 43 | Chamomile | *Ormenis mixta* | flower | *Asteraceae* | Morocco |
| 44 | Ay tsao | *Artemisia argyi* | leaf | *Asteraceae* | China |
| 45 | Lemongrass | *Cymbopogon citratus* | leaf | *Poaceae* | India |
| 46 | Vetiver | *Vetiveria zizanoides* | root and leaf | *Poaceae* | Haiti |
| 47 | Citronella ceylon | *Cymbopogon nardus* | seed | *Poaceae* | China |
| 48 | Myrrh | *Commiphora myrrha* | branch | *Commiphora* | Bengal |
| 49 | Frankincense | *Boswellia thurifera* | bark | *Burseraceae* | Turkey |
| 50 | Marjoram | *Origanum marjorana* | flower and leaf | *Lamiaceae* | Bulgaria |
| 51 | Thyme | *Thymus vulgaris* | entire plants | *Lamiaceae* | Spain |
| 52 | Clary sage | *Salvia sclarea* | flower and leaf | *Lamiaceae* | France |
| 53 | Peppermint2 | *Mentha haplocalyx Briq* | leaf | *Lamiaceae* | America |
| 54 | Melissa | *Melissa officinalis* | flower and leaf | *Lamiaceae* | France |
| 55 | Peppermint1 | *Mentha piperita* | leaf | *Lamiaceae* | America |
| 56 | Basil | *Ocimum basilicum* | leaf | *Lamiaceae* | Egypt |
| 57 | Patchouli | *Pogostemon patchouli* | entire plants | *Lamiaceae* | India |
| 58 | Rosemary | *Rosmarinus officinalis* | flower and leaf | *Lamiaceae* | Spain |
| 59 | Juniper | *Juniperus communis* | fruit | *Cupressaceae* | Spain |
| 60 | Cypress | *Cupressus sempervirens* | leaf and cone | *Cupressaceae* | England |

**Table S2.** Repelling rate of 60 EOs at 10 μg/cm$^2$ exposed for 30 min

| EO | 1 | 2 | 3 | Average | SD |
|---|---|---|---|---|---|
| Cinnamon | 78% | 80% | 72% | 77% | 4% |
| Marjoram | 59% | 55% | 56% | 57% | 2% |
| Lemongrass | 50% | 56% | 56% | 54% | 3% |
| Bay | 50% | 52% | 50% | 51% | 1% |
| Chamomile | 49% | 44% | 47% | 47% | 3% |
| Jasmine | 47% | 40% | 45% | 44% | 4% |
| Peppermint2 | 30% | 50% | 45% | 42% | 10% |
| Thyme | 39% | 43% | 40% | 41% | 2% |
| Osmanthus | 42% | 33% | 33% | 36% | 5% |
| Myrrh | 32% | 34% | 34% | 33% | 1% |
| Melissa | 37% | 28% | 33% | 33% | 5% |
| Grapefruit | 33% | 28% | 32% | 31% | 3% |
| Sandalwood | 34% | 33% | 27% | 31% | 4% |
| May Chang | 28% | 31% | 32% | 30% | 2% |
| Citronella ceylon | 33% | 27% | 28% | 29% | 3% |
| Nutmeg | 32% | 26% | 28% | 29% | 3% |
| Vetiver | 26% | 28% | 31% | 28% | 3% |
| Ay tsao | 22% | 24% | 32% | 26% | 5% |
| Bergamot | 33% | 18% | 25% | 25% | 8% |
| Clary sage | 24% | 18% | 33% | 25% | 8% |
| Petitgrain | 26% | 22% | 24% | 24% | 2% |
| Green tea | 26% | 25% | 19% | 23% | 4% |
| Geranium | 21% | 28% | 20% | 23% | 4% |
| Verbena | 28% | 21% | 17% | 22% | 6% |
| Benzoin | 23% | 19% | 20% | 21% | 2% |
| Peppermint1 | 17% | 21% | 22% | 20% | 3% |
| Clove | 16% | 22% | 18% | 19% | 3% |
| Michelia alba flower | 15% | 20% | 23% | 19% | 4% |
| Basil | 25% | 11% | 18% | 18% | 7% |
| Patchouli | 15% | 20% | 15% | 17% | 3% |
| Neroli | 21% | 11% | 18% | 17% | 5% |
| Cedarwood | 26% | 8% | 15% | 16% | 9% |
| Palmarosa | 17% | 13% | 14% | 15% | 2% |
| Michelia alba leaf | 21% | 11% | 13% | 15% | 5% |
| Lime | 11% | 16% | 15% | 14% | 3% |
| Rosemary | 11% | 12% | 15% | 13% | 2% |
| Parsley | 22% | 6% | 12% | 13% | 8% |
| Juniper | 17% | 13% | 6% | 12% | 6% |
| Lemon eucalyptus | 9% | 10% | 13% | 11% | 2% |
| Capsicum | 15% | 6% | 8% | 10% | 5% |
| Angelica | 5% | 13% | 10% | 9% | 4% |

| | | | | | |
|---|---|---|---|---|---|
| Cajeput | 5% | 11% | 12% | 9% | 4% |
| Orange sweet | 11% | 6% | 8% | 8% | 3% |
| Carrot seed | 14% | 4% | 6% | 8% | 5% |
| Eucalyptus | 0% | 11% | 6% | 6% | 6% |
| Tangerine | 5% | 5% | 5% | 5% | 0% |
| Violet | 0% | 6% | 2% | 3% | 3% |
| Fennel | 0% | 6% | 3% | 3% | 3% |
| Rose | -7% | 12% | 4% | 3% | 10% |
| Ginger | 7% | 0% | 0% | 2% | 4% |
| Ylang ylang | 0% | 1% | 3% | 1% | 2% |
| Chinese ilex | -4% | 0% | 6% | 1% | 5% |
| Cypress | 0% | 0% | 1% | 0% | 1% |
| Black pepper | 5% | -12% | 5% | -1% | 10% |
| Cumin | -5% | 0% | 0% | -2% | 3% |
| Pine fir | -11% | -5% | 0% | -5% | 6% |
| Mandarin | -7% | -6% | -5% | -6% | 1% |
| Rosewood | -7% | -8% | -5% | -7% | 2% |
| Frankincense | -20% | -10% | 0% | -10% | 10% |
| Lemon | -20% | -6% | -9% | -12% | 7% |
| DEET | 55% | 60% | 61% | 59% | 3% |

**Table S3.** Repelling rate of the main constituents from 8 active EOs

| Compounds | 1 | 2 | 3 | Average | SD |
|---|---|---|---|---|---|
| cinnamaldehyde (CZ, LN) | 79% | 84% | 82% | 82% | 3% |
| citral (CC) | 67% | 60% | 68% | 65% | 4% |
| terpinen-4-ol (OMa) | 59% | 64% | 56% | 60% | 4% |
| thymol (TV) | 60% | 55% | 44% | 53% | 8% |
| benzyl acetate (JS) | 34% | 38% | 38% | 37% | 2% |
| diethyl phthalate (OMi) | 30% | 40% | 41% | 37% | 6% |
| eugenol (LN) | 35% | 31% | 36% | 34% | 3% |
| diisobutyl phthalate (LN) | 41% | 31% | 23% | 32% | 9% |
| $\beta$-caryophyllene (LN) | 28% | 29% | 34% | 30% | 3% |
| $\alpha$-terpineol (OMa) | 20% | 24% | 25% | 23% | 3% |
| menthol (MHB) | 16% | 23% | 19% | 19% | 4% |
| carvacrol (TV) | 18% | 19% | 14% | 17% | 3% |
| linalool (JS, TV) | 20% | 11% | 13% | 15% | 5% |
| limonene (OMa, CC, OMi, MHB, TV) | 9% | 16% | 14% | 13% | 4% |
| *p*-menthone (MHB) | 3% | 4% | 6% | 4% | 2% |
| (*E*)-2-hexyl-cinnamaldehyde (JS) | 0% | 3% | 3% | 2% | 2% |
| 2,2,4,6,6-pentamethyl-heptane (MHB) | -3% | -3% | 4% | -1% | 4% |
| $\gamma$-terpinene (OMa) | 0% | -14% | -9% | -8% | 7% |
| *p*-cymene (OMa, TV) | -10% | -16% | -5% | -10% | 6% |
